# The mitochondrial outer-membrane location of the EXD2 exonuclease contradicts its direct role in nuclear DNA repair

**DOI:** 10.1038/s41598-018-23690-y

**Published:** 2018-03-29

**Authors:** Fenna Hensen, Amandine Moretton, Selma van Esveld, Géraldine Farge, Johannes N. Spelbrink

**Affiliations:** 1 Radboud Center for Mitochondrial Medicine, Department of Paediatrics, Radboudumc, Nijmegen The Netherlands; 2 Université Clermont Auvergne, CNRS/IN2P3, Laboratoire de Physique de Clermont, BP 10448, F-63000 Clermont-Ferrand, France; 3 Radboud Center for Mitochondrial Medicine & Center for Molecular and Biomolecular Informatics, Radboud Institute for Molecular Life Sciences, Radboudumc, Nijmegen The Netherlands

## Abstract

EXD2 is a recently identified exonuclease that has been implicated in nuclear double-strand break repair. Given our long standing interest in mitochondrial DNA maintenance and indications that EXD2 could also be a mitochondrial protein we sought to determine its cellular localization and possible mitochondrial associated functions. Our results show that EXD2 indeed shows mitochondrial localization, but, surprisingly, is found predominantly associated with the mitochondrial outer-membrane. Gradient purified nuclei show only the faintest hint of EXD2 presence while overexpression of the predicted full-length protein shows exclusive mitochondrial localization. Importantly, induction of double-strand DNA breaks via X-irradiation or Zeocin treatment does not support the notion that EXD2 re-locates to the nucleus following double-strand breaks and thus is unlikely to have a direct role in nuclear DNA repair. Knockdown or overexpression of EXD2 affects the cellular distribution of mitochondria. These results suggest that the reported defects in nuclear DNA repair following EXD2 depletion are likely an indirect consequence of altered mitochondrial dynamics and/or function.

## Introduction

Protein function can often be predicted on the basis of signature amino-acid motifs. Exonucleases are no exception to this rule. However, although a bioinformatics prediction for function in some cases can be unquestionable and *in vitro* activity measurements verify predicted enzymatic activity, if the protein in question is not located in the compartment where it is supposed to act, one has to reconsider its function. EXD2 is a newly identified exonuclease that has recently been implicated in nuclear double-strand break repair^1–3^. We have a long standing interest in mtDNA maintenance enzymes including nucleases^4,5^, and as more and more nuclear DNA maintenance proteins have in recent years been assigned a mitochondrial function, we have a keen interest in newly discovered nuclear enzymes. A closer inspection of various available online databases and tools showed that despite its recent proposed role in nuclear DNA repair, EXD2 location is predicted to be mitochondrial/cytoplasmic. Cellular and mitochondrial localization prediction programs vary in their estimation. For example MitoProt II^6^ gives a reasonably high mitochondrial probability score of 69%, PSORT II^7^ gives a poor mitochondrial prediction and TargetP^8^ suggests the protein is secreted. Several published papers have suggested a mitochondrial function for EXD2 (Mason and Cox^9^ and references herein). Most striking however is that the antibody used both by Broderick *et al*. and Biehs *et al*.^1,2^ is reported at http://www.proteinatlas.org ^10^ as showing a distinct mitochondrial and possibly intermediate filament localization using immunofluorescence (IF). For this reason, we here have re-examined the subcellular localization and function of EXD2.

## Results and Discussion

### The main cellular localization of EXD2 is the mitochondrial outer membrane

**Figure 1:**
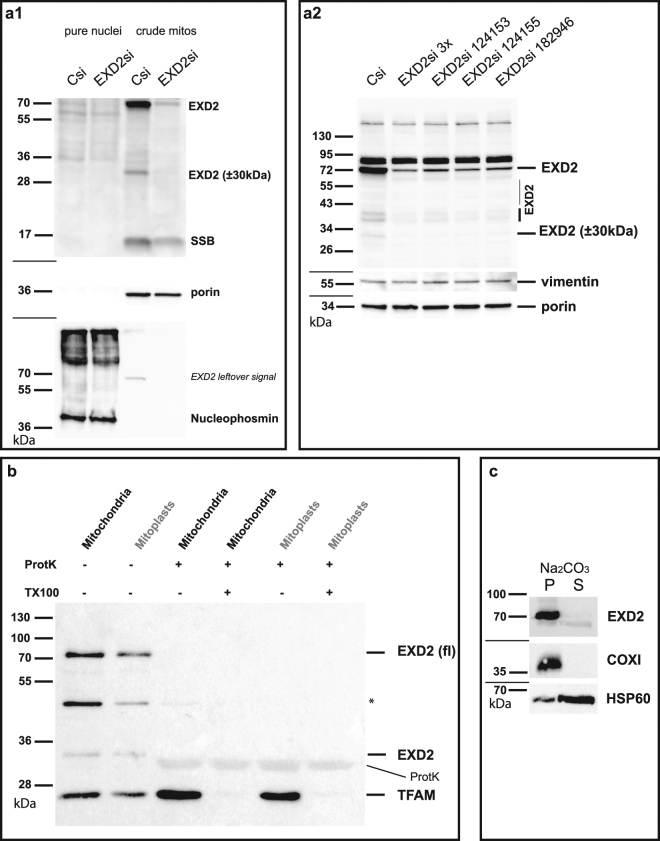
EXD2 is a mitochondrial outer-membrane associated protein. U2OS cell crude mitochondria isolated by differential centrifugation and pure nuclei isolated on iodixanol gradients were tested for EXD2 abundance in control siRNA and EXD2 siRNA treated cells (panel a1). Results show that the vast majority of EXD2, similar to mitochondrial marker proteins mtSSB and porin are found in the mitochondrial fraction and not in the nuclear fraction, in which the nuclear marker nucleophosmin is identified. SiRNA treatment confirms the identity of the full length (fl) ~70 kDa EXD2 protein and several lower abundant species of 30 kDa and higher molecular weight. Knockdown with either the combined pool of three commercial siRNAs or each individual siRNA show similar knockdown efficiencies in total cell lysates of U2OS cells on Western blots (panel a2). At the same time, several lower molecular weight EXD2 species are also identified and all appear to be equally sensitive to each individual siRNA suggesting they might be breakdown or processed EXD2 forms. Protease protection demonstrates mitochondrial EXD2 is mostly found in the mitochondrial outer-membrane (panel b). Mitochondria and digitonin-derived mitoplasts from HEK293 cells were treated either with Proteinase K (ProtK) alone or with ProtK and Triton-X100 (TX100) to lyse the inner and outer membrane. Results show that whereas TFAM, an mtDNA associated protein, is protected from ProtK in the absence of TX100, EXD2 is not, suggesting and outer-membrane localization. *Indicates a remnant signal from the probing with an antibody against a different mitochondrial candidate protein not relevant for this paper. A Na_2_CO_3_ extract of crude mitochondria from HEK293 cells (panel c) shows that similar to cytochrome *c* oxidase subunit I (an integral membrane protein), full length EXD2 is found predominantly in the pellet (membrane) fraction, whereas the majority of HSP60 is found in the supernatant (non-membrane) fraction. For each panel (except panel b) cropped images show the results of incubations with subsequent antibodies on the same blots, indicated by dividing lines (see Supplementary info for full blot images).

We used biochemical fractionation experiments and IF in combination with EXD2 knockdown or overexpression to establish its subcellular localization. Crude mitochondrial fractions of control and EXD2 siRNA treated U2OS cells clearly identified distinct EXD2 bands, the most prominent one corresponding to the predicted full-length protein of approximately 70 kDa, a second weaker species of approximately 30 kDa (Fig. 1 panel a1 and a2) and several even fainter bands between 70 and 30 kDa. Knockdown with three individual siRNAs, instead of a mix of all three that we more routinely use (see M&M), showed similar knockdown of all detected bands with each siRNA (Fig. 1, panel a2). Since the three siRNA’s cover almost 1000 bp of the 1866 bp open reading frame, it seems likely that the smaller forms are either processed forms or breakdown products.

**Figure 2:**
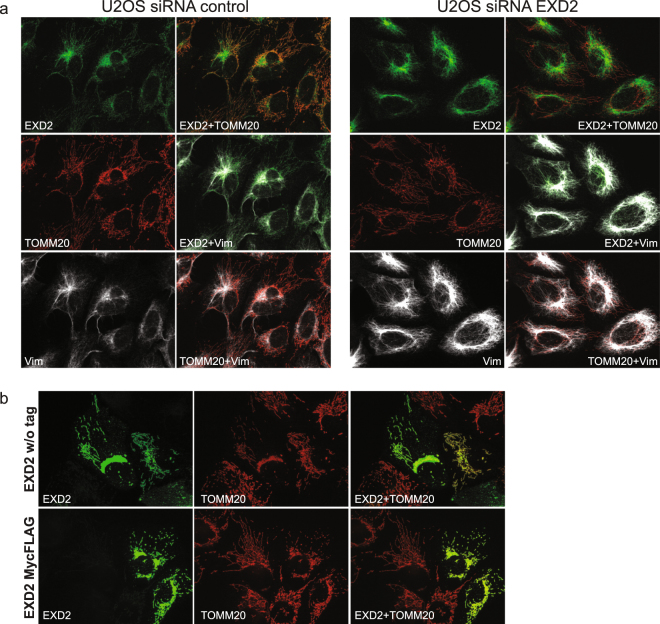
Knockdown or overexpression in U2OS cells of full-length EXD2 confirms the mitochondrial localization of EXD2. ProteinAtlas describes their EXD2 antibody, which we have used throughout this study, as having a mitochondrial and possible intermediate filament localization. To test the localization and the validity of their antibody we tested the EXD2 antibody, together with an antibody against the outer-membrane protein Tomm20 and an antibody against the intermediate filament protein vimentin (Vim) using immunofluorescence following transfection with either a pool of non-targeting control siRNAs or a pool of three EXD2 Stealth siRNAs (panel a). Co-staining in control siRNA cells with Tomm20 and vimentin shows co-localization of the EXD2 signal both with mitochondrial and intermediate filament signals. EXD2 siRNA treatment shows that while the EXD2 mitochondrial signal is no longer observed, the intermediate filament signal remains suggesting that this signal is either non-specific or that the siRNA pool used does not affect intermediate filament associated EXD2. Transient overexpression of the predicted full length protein, either w/o a tag or with a C-terminal combined Myc/FLAG tag shows an exclusive mitochondrial localization of the protein as illustrated by Tomm20 co-staining, while higher level overexpression results in mitochondrial perinuclear clustering (panel b). With very high overexpression, the whole mitochondrial network collapsed in one large perinuclear cluster that had lost any typical mitochondrial network-like structure (Supplementary Fig. S1). Overexpressed full length EXD2 showed no evidence of either nuclear or intermediate filament localization. Please note that for this Figure images have been selected deliberately to best illustrate the mitochondrial localization of EXD2. Image views have thus been chosen showing cells with a clear and extended mitochondrial network, while trying to avoid cells with a condensed/collapsed mitochondrial network.

The nuclear pellets obtained during the crude mitochondrial fractionation were further purified using iodixanol gradient purification to remove excess mitochondria from the nuclear fractions^11,12^, and ran alongside the mitochondrial fractions (Fig. 1a1). Probing with the EXD2 antibody clearly shows that the vast majority of EXD2 is found in the mitochondrial fraction and not in the nuclear fraction (the same fractionation results were obtained using HEK293 cells, not shown). Control antibodies exclude major nuclear or mitochondrial contamination of the mitochondrial and nuclear fraction, respectively. Nonetheless, a faint band for full-length EXD2 is observed in the nuclear fraction, but likewise mtSSB shows a faint nuclear signal, suggesting a minor mitochondrial contamination of this fraction. This is further corroborated by the observation that neither IF nor IF following overexpression of the full-length protein shows evidence for nuclear EXD2 (see below, Fig. 2).

The predicted function of EXD2 combined with a mitochondrial localization suggested it might be involved in mtDNA metabolism. Examination of EXD2 mitochondrial localization using proteinase K (protK) protection showed that the protein was sensitive to digestion using intact mitochondria and outer-membrane-lysed mitochondria (here referred to as mitoplasts, see^5^), without the addition of Triton-X100 (TX100) to fully lyse mitochondria (Fig. 1b). In contrast, transcription factor A of mitochondria (TFAM), a well-known mtDNA binding protein that is essential for mtDNA maintenance^13^, was insensitive to externally added protK and required the addition of TX100 for its proteolysis. These data point to an outer-membrane localization of EXD2 and seem to exclude an mtDNA maintenance function. Online membrane-spanning amino-acid (AA) sequence prediction tools suggest a single trans-membrane region at the N-terminus of the full-length 621 AA protein. A tight membrane association is supported by Na_2_CO_3_ extraction of crude mitochondria (Fig. 1c), in which case EXD2 fractioned to the pellet together with for example the integral membrane protein COXI, whereas the majority of HSP60, a matrix protein, was found in the supernatant.

To further examine EXD2 localization, we used IF. Human ProteinAtlas shows a clear mitochondrial and a possible intermediate filament EXD2 localization with the ProteinAtlas antibody that we have used also here. However, since this antibody has not been validated for IF, we re-examined these findings, using siRNA mediated knockdown. In addition we used transient overexpression of the predicted full-length protein to examine EXD2 localization using a validated cDNA clone. IF with the EXD2 antibody and the mitochondrial outer membrane marker Tomm20, showed a clear mitochondrial EXD2 signal in non-transfected U2OS cells (Fig. 2a) or primary fibroblasts (not shown) and in addition a signal reminiscent of intermediate filaments. This was confirmed by a co-incidental signal with a vimentin antibody. SiRNA mediated knockdown of EXD2 depleted the mitochondrial signal, however the intermediate filament signal remained, suggesting either a non-specific antibody cross reactivity with intermediate filaments or an EXD2 protein isoform that is not or poorly targeted by our siRNA pool, or much more stable and therefore less prone to siRNA mediated depletion. Using IF, again no evidence supported the presence of EXD2 in the nucleus.

**Figure 3:**
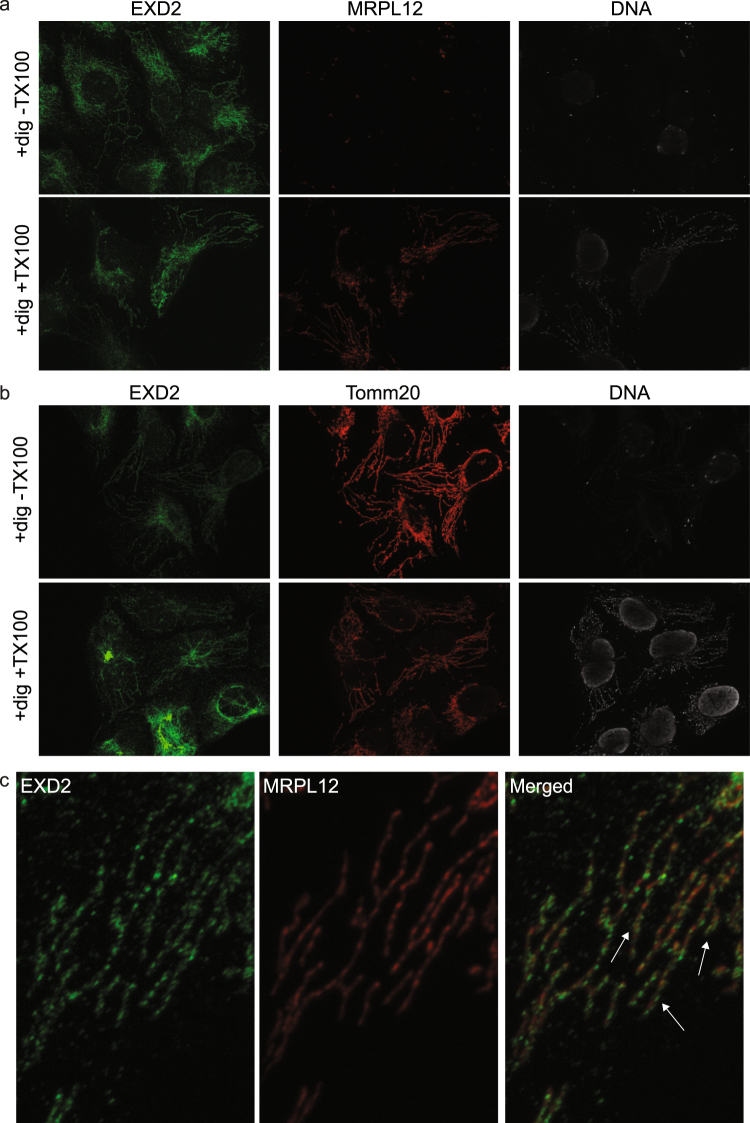
EXD2 is accessible to added antibody in immunofluorescence without mitochondrial lysis. Immunofluorescent detection following paraformaldehyde fixation requires mitochondrial lysis using for example Triton X100. In the absence of this lysis step mitochondrial matrix proteins and for example mtDNA are not detectable. Thus (panel a) results shows that in the absence of TX100 lysis, EXD2 is detected while neither MRPL12 nor mtDNA are detected by IF. With TX100 lysis, all three are detected. A similar experiment (panel b) shows that both Tomm20 and EXD2, but not mtDNA are detected in the absence of TX100 lysis. A high resolution 20 × 30 µM subsection of a cell using the EXD2 and MRPL12 antibodies illustrates that the EXD2 signal is often enveloping the MRPL12 signal (panel c: some examples are indicated by a white arrow in the merged image), further illustrating EXD2 its outer-membrane localization.

While this paper was under review, the mitochondrial localization of EXD2 was also reported by others^14^. However, a very crucial difference between this paper and our results concerns the localization of EXD2. While Silva *et al*. suggest EXD2 is predominantly located in the mitochondrial matrix and has a function in mitochondrial translation, we suggest the protein is located in the mitochondrial outer membrane. Since, the protK protection assay as presented in Fig. 1 is not all conclusive because it for example depends on a protein’s sensitivity to proteolysis, we further addressed this issue of contention using an *in situ* protein accessibility assay combined with IF. This assay is based on the fluorescent protease protection (FPP) assay^15^ and uses increasingly stringent lysis conditions to make the mitochondrial matrix accessible to proteins. Whereas in the case of the original FPP assay the sensitivity of e.g. GFP tagged proteins to added protK is tested, we here use the EXD2 antibody and control antibodies to test for antibody accessibility to the protein of interest. Following paraformaldehyde fixation and lysis with digitonin only, mitochondrial matrix proteins are inaccessible to externally added proteins such as antibodies and therefore not detectable using IF. If in contrast cells are lysed with TX100 following digitonin lysis, full antibody accessibility ensures detection of all proteins including matrix proteins. Thus, using this assay, we show that in the absence of TX100, the mitochondrial matrix protein MRPL12 (also used by Silva *et al*. in a co-localization assay)^14^ and mtDNA are not detected. However EXD2 is as easily detected as in the presence of TX100 (Fig. 3a). In a second series we used the outer membrane marker Tomm20 and show that it, as well as EXD2, but not mtDNA are readily detected in the absence of TX100 (Fig. 3b). These results thus clearly show that EXD2 is either outer-membrane localized or, alternatively embedded in the inner membrane with its single N-terminal transmembrane domain and the rest of the protein facing out, given that the commercial antibody has its epitope in the middle of the protein. However, careful examination of the obtained images in this experiment at high magnification does suggest the correct localization is in fact the outer membrane. Without TX100 lysis, both EXD2 and Tomm20 images show a broader mitochondrial signal than typically seen for mitochondrial matrix proteins, sometimes suggesting the appearance of a hollow tube. This is confirmed by the images using combined digitonin and TX100 lysis and detection of EXD2 and MRPL12, suggesting a narrower signal for MRPL12, with the EXD2 signal often clearly enveloping the MRPL12 signal (a 20 × 30 µm cellular subsection illustrates this in Fig. 3c). In conclusion, both our biochemical and immunofluorescence assay as well as high resolution imaging of EXD2 and the mitochondrial matrix protein MRPL12 consistently support an outer-membrane localization of EXD2.

### EXD2 overexpression or knockdown suggest an involvement in mitochondrial morphology and/or dynamics

**Figure 4:**
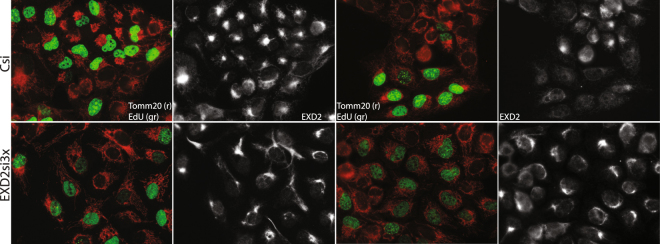
SiRNA mediated knockdown of EXD2 in U2OS cells results in a more widely spread mitochondrial network. In order to understand possible effects of EXD2 knockdown on mitochondrial network behaviour we labelled cells with EdU to identify cells in S-phase, in order to get a handle on mosaicism resulting from cell-cycle mediated effects on mitochondrial network dynamics (see main text). For this, cells were incubated for 30 min with EdU following transfection and 48–60 hrs incubation with either a pool of non-targeting control siRNAs (Csi) or a pool of three EXD2 stealth siRNAs (EXD2si3x). Following labelling for EdU incorporation (green, gr), cells were further labelled with antibodies for Tomm20 (red, r) and EXD2 (white). Control siRNA treated cells show frequent and strong perinuclear clustering of the mitochondrial network in particular in EdU positive cells. In contrast, the EXD2si3x typically show a more distributed and sometimes more hyperfused network. Two fields of view are shown for each condition.

Overexpression of either a non-tagged or C-terminally tagged predicted full-length version of the protein showed exclusive mitochondrial localization (Fig. 2b). High-level overexpression (based on IF signal) of both the non-tagged and tagged variant resulted in abnormal mitochondrial network morphology with extreme perinuclear clustering (multiple fields of view of non-tagged transiently overexpressed EXD2 are presented in Supplemental Fig. S1, showing the collapse of the mitochondrial network with increasing EXD2 expression). In contrast, siRNA mediated knockdown suggested a more distributed mitochondrial network, with fewer cells compared to control siRNA-treated cells showing perinuclear mitochondrial abundance. These data might suggest a mitochondrial dynamics/morphology function of EXD2 (Fig. 4 and Supplementary Fig S1).

Mitochondrial dynamics and morphology is in part cell cycle dependent^16^, resulting in mosaic patterns of mitochondrial-cellular distribution and morphology in cultured cells. For this reason and to more clearly assess the EXD2 knockdown phenotypes in relation to the EXD2 antibody intermediate filament co-staining, we repeated knockdown experiments. In control siRNA cells, a considerable proportion showed very localized perinuclear clustering of the mitochondrial network, in particular in those cells that are also positive for EdU nuclear DNA incorporation, showing that cells are in S-phase (Fig. 4). In contrast, with the combined siRNA pool (EXD2si3x) we observed a more distributed mitochondrial network. This was not a variable response to EdU labelling as the same is observed in cells not labelled using EdU (see Supplementary Fig. S1). As also observed in Fig. 2, intermediate filament staining by the EXD2 antibody did not appear to be affected by the knockdown. In addition, vimentin antibody staining of Western blots following knockdown, did not show reduced vimentin levels (see Fig. 1, panel a2). Although at this point we cannot completely exclude a direct EXD2-intermediate filament interaction, our data suggest no effect of EXD2 knockdown on vimentin. In addition, overexpression of the full-length protein showed no evidence for intermediate filament co-localization (Fig. 2b). To summarize, EXD2 is mainly embedded in the mitochondrial outer membrane and its overexpression or knockdown both appear to affect mitochondrial network morphology/dynamics.

### Induction of nuclear DNA damage does not result in nuclear translocation of EXD2

**Figure 5:**
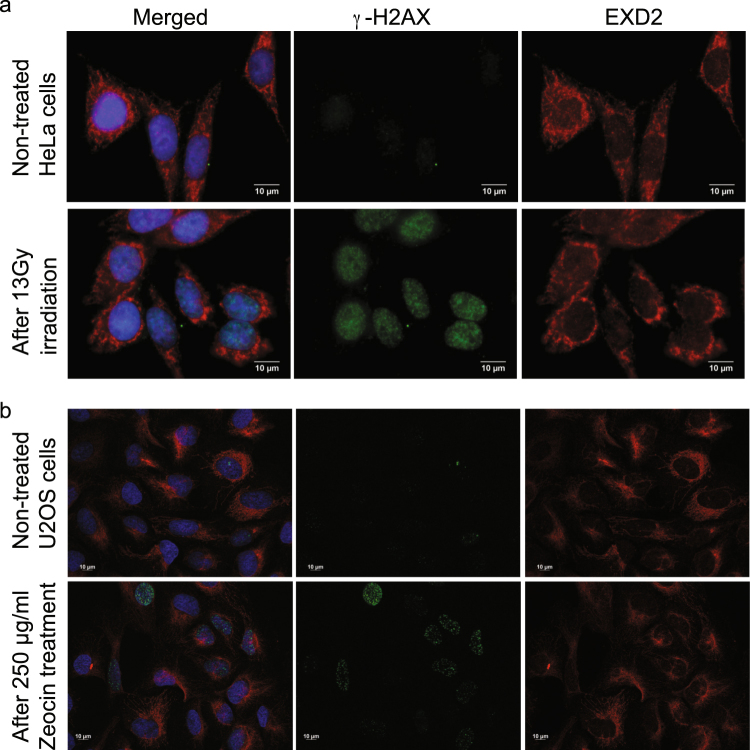
Nuclear DNA-damage does not relocate EXD2 to the nucleus. (panel a) HeLa cells were treated with 13 Gy X-irradiation to induce nuclear DNA damage and allowed to recover for 30 minutes. Results show that H2AX phosphorylation is considerably increased in the nucleus following this damage whereas EXD2 retains its predominant mitochondrial localization similar to its location in control cells. (panel b) U2OS cells were treated with 250 µg/ml Zeocin for 1 hr and allowed to recover for 1 hr after replacing the Zeocin-containing medium with regular medium. Cells were then processed for IF detection of γH2AX and EXD2 and show a similar result as the X-irradiation in HeLa cells.

Broderick *et al*.^2^, have stated that ‘EXD2 is recruited to chromatin in a damage dependent manner’, yet they have not shown this directly in their paper. Since the main localization of EXD2 is the mitochondrial outer membrane we hypothesized that upon nuclear DNA damage, EXD2 might perhaps relocate to the nucleus. To address this possibility we treated HeLa cells (also used by Broderick *et al*.) with ionizing radiation (Fig. 5). Even though the DNA-damage γH2AX marker (e.g. Burma *et al*.)^17^ showed a strong enhancement of its nuclear signal following this treatment, EXD2 showed no re-distribution upon ionizing radiation, thus showing no evidence for a direct damage-foci localized function in nuclear DNA repair. Similarly, treatment of U2OS cells with Zeocin (a Bleomycin antibiotic family member) showed considerable enhancement of the γH2AX nuclear signal, while it had no effect on EXD2 mitochondrial outer-membrane localization.

To conclude, while it has been attractive based on available data to implicate EXD2 as a new player in nuclear double-strand break-repair^3^, we here show that EXD2 is a relatively abundant outer-mitochondrial-membrane protein. For the various cells lines we have tested including HeLa cells there is very little if any evidence that the protein is also nuclear, either under normal conditions or following the induction of nuclear DNA damage. This is in agreement with cellular localization predictions, online antibody data from ProteinAtlas and also a recent paper using proximity biotinylation of mitochondrial outer membrane and ER membrane proteins^18^, results of which also support an outer mitochondrial membrane and possibly ER-mitochondrial contact-site localization of EXD2. Our results also contradict a mitochondrial matrix localization for EXD2, as was recently reported^14^ and therefore do not support a direct role in mitochondrial translation. In contrast, EXD2 seems to play a role in mitochondrial dynamics, which in turn could explain the effects of its knockdown or knockout on nuclear DNA replication and/or repair (see e.g. Qin *et al*.)^19^, yet again illustrating the importance of mitochondrial dynamics in diverse cellular processes. In the future, we hope to further clarify the exact molecular function of EXD2 by identification of its interaction partners using proximity labelling/mass spectrometry methods and clarify a possible ER interaction.

## Materials and Methods

### Cell culture

HeLa, HEK293 (ATCC CRL-1573), U2OS cells (University of Helsinki, Finland) and in-house primary human fibroblasts were grown in Dulbecco’s modified Eagle’s medium (DMEM; Lonza BE12-604F) supplemented with 10% fetal calf serum (FCS) (GE Healthcare), in a 37 °C incubator at 5% CO_2_. All cell lines were routinely tested for mycoplasma contamination and found to be negative.

#### RNA-interference and overexpression

For EXD2 knockdown, cells were transfected in 6 well plates (for IF) or 10 cm cell culture dishes (for biochemical fractionation experiments) with a mixture of three Stealth^TM^ siRNA duplex oligonucleotides (EXD2 HSS124153, HSS124155 and HSS182946 Invitrogen), at a concentration of 10 nM each, using Lipofectamine^TM^2000. Alternatively the individual siRNAs were used at a concentration of 30 nM. As controls we used Stealth^TM^ Universal negative controls at the same concentrations.

A commercial cDNA clone for the predicted full-length cDNA for EXD2 and containing a Myc-DDK (FLAG) tag was acquired from Origene (Catalogue nr. RC231340) and recloned either with or without tag in a gateway customized pcDNA5/FRT/TO (Invitrogen) plasmid (kind gift of Dr. LGJ Nijtmans) using the Gateway recombination system. Inserts were fully sequenced to verify the wild-type sequence. Plasmids were used in transient transfections using TransIT-LT1 transfection reagent (Mirus), using manufacturer’s instructions.

#### Mitochondrial isolation and iodixanol gradient purification of nuclei

U2OS cells were collected and resuspended in hypotonic buffer (4 mM Tris-HCl, pH 7.8, 2.5 mM NaCl, 0.5 mM MgCl_2_ including protease inhibitors (Roche Molecular Biochemicals)) allowed to swell for 10 min and disrupted with 20–25 strokes with a Dounce homogenizer on ice. The suspension was re-isotonised with 0,1 volume of 400 mM Tris-HCl, 250 mM NaCl and 50 mM MgCl_2_. Nuclei and cell debris were first pelleted by centrifugation at 1 200 *g* for 5 min at 4 °C. The low speed centrifugation was repeated once and crude mitochondria were then pelleted from the cytosol by centrifugation at 13 000 *g* for 10 min at 4 °C. The low speed nuclear pellet was further subjected to iodixanol gradient purification as previously described^11^ and pure nuclei were extracted in 20 mM Tris/HCl (pH 7.9), 25% glycerol, 0.42 M NaCl, 1.5 mM MgCl_2_, 0.2 mM EDTA, 0.1 mM PMSF and 0.5 mM DTT^20^. Na_2_CO_3_ extraction of crude mitochondria was done as previously described^5^.

#### Protein analyses

Were carried out by Western blotting after separation by SDS-PAGE. Proteins were transferred from the gel onto a nitrocellulose membrane, which was probed with antibodies against proteins of interest and HRP-conjugated secondary antibodies followed by ECL detection. ECL reactions were visualized with a ChemiDoc instrument (Biorad). Antibodies used for Western blot detection were mtSSB (Sigma, HPA002866), EXD2 (Sigma, HPA005848), vimentin (Abcam Ab24525), nucleophosmin (Invitrogen, 32–5200), Porin (Invitrogen, A31855), TFAM (kind gift of Dr. R. Wiesner), COXI (Invitrogen), HSP60 (Abcam, Ab46798).

### Immunofluorescence

For immunofluorescent detection cells were grown on coverslips in 6 well plates. Cells were fixed using 3.3% paraformaldehyde (PFA) in cell culture medium for 15–25 min, washed 3x in PBS and permeabilized for 15 min with 0.5% Triton X100 in PBS/10% FCS. Primary and secondary antibodies were incubated at the following concentrations in PBS/10%FCS for 1 h: EXD2 rabbit polyclonal (Sigma, HPA005848), 1:200; Tomm20 mouse monoclonal (Santa Cruz, sc17764), 1:100; MRPL12 mouse monoclonal (Abcam, Ab58334), 1:200; vimentin chicken polyclonal (Abcam, Ab24525), 1:200. Secondary goat-anti-rabbit, goat-anti-mouse and goat anti-chicken IgG were AlexaFluor 488, 568, and 647 (Invitrogen) labelled and used in various combinations at a 1:1000 dilution. 5-ethynyl-2′-deoxyuridine (EdU) labelling and detection (Invitrogen), to identify nuclear DNA synthesis, was done as previously described^5^ with in this case a short 30 min labelling period. For the immunofluorescent antibody accessibility assay, cells were treated with 100 µM digitonin in PBS for 7 min at RT following PFA fixation. Cells were subsequently washed 3x with PBS and either or not further permeabilized with TX100 followed by antibody incubations as above. For U2OS Zeocin^TM^ (Invivogen) treatment, Zeocin was added at a final concentration of 250 µg/ml and incubated for 1 hr. Cells were washed once with cell culture medium and allowed to recover for an additional hour in cell culture medium. Slides were then processed for immunofluorescent detection with EXD2 and gamma-H2AX antibodies (see below: Nuclear DNA damage and repair induction by irradiation). Slides were mounted using ProLong® Gold antifade with DAPI (Invitrogen). Image acquisition used the Zeiss apotome system on an axio-observer Z.1 with Colibri led illumination and appropriate emission filters. In all cases in which controls are compared with experimental manipulation of cells (for example using siRNA), images have been acquisitioned with identical illumination and exposure settings and processed identically. Where needed, images were further processed using ImageJ or Photoshop to adjust brightness/contrast and size/resolution.

### Nuclear DNA damage and repair induction by irradiation

HeLa cells were grown overnight on coverslips in 6 well plates and X-irradiated with doses of 0 or 13 Gy with an X-RAD 320 biological irradiator (Precision X-ray, Inc., PAVIRMA platform, Clermont-Ferrand, France). After a 30 min recovery at 37 °C, cells were washed with PBS, fixed with 3.7% formaldehyde in PBS at 37 °C for 15 min, washed 3x with PBS-0.1% Tween (PBST), permeabilized with PBS-0.5% Triton 100× at RT for 10 min, washed 3 times with PBST and incubated with a blocking solution (1% BSA, 22.52 mg/mL glycine in PBST) at RT for 30 min. For visualization of EXD2 and gamma-H2AX, coverslips were then incubated with anti-EXD2 antibody (1:200 in PBS, Sigma HPA005848) and anti-gamma-H2AX antibody (1:200 in PBS, Merck Millipore 05-636-I) at RT for 1 hr30. Unbound antibodies were removed with 3 washes with PBST. Coverslips were incubated at RT for 1hr with secondary antibodies, anti-rabbit IgG conjugated to Alexa Fluor 555 (1:1000 in PBS) and anti-mouse IgG conjugated to Alexa Fluor 488 (1:1000 in PBS, Molecular Probes A21151). Cells were washed 3x with PBST and DNA was counterstained with DAPI. A final wash was performed with PBS. Coverslips were mounted using 10ul PBS/Glycerol (50/50, v/v) and observed using a Leica MMAF Imaging NX microscope powered by Metamorph.

### Data availability

The data generated during and/or analyzed during the current study are available from the corresponding author on reasonable request.

## Electronic supplementary material

Supplementary Figure 1
