# Supplementary material for: The mitochondrial outer-membrane location of the EXD2 exonuclease contradicts its direct role in nuclear DNA repair
Source: Sci Rep. 2018 Mar 29;8:5368. doi: 10.1038/s41598-018-23690-y (PMC5876329; doi:10.1038/s41598-018-23690-y)
Supplement: Supplementary file 1 — Supplementary Figure 1 [file 41598_2018_23690_MOESM1_ESM.pdf]

The mitochondrial outer-membrane location of the EXD2 exonuclease contradicts its direct role in nuclear DNA repair

Fenna Hensen, Amandine Moretton, Selma van Esveld, Géraldine Farge & Johannes N. Spelbrink

Supplementary information

Supplementary Fig S1 Legend:
U2OS cells were treated either with control (top row) or EXD2 siRNA (second row) and processed for immunofluorescence 48 hrs after transfection. For both treatments three fields of views are shown as merged fluorescence images with EXD2 antibody staining shown in green, Tomm20 in red and DAPI in blue. Bottom two rows show three panels of U2OS cells transiently transfected for 48 hrs with an non-tagged EXD2 expression construct (see also Fig 2). Shown are merged fluorescent images showing EXD2 in green, Tomm20 in red and DAPI in blue (third row of panels), while the fourth row of panels shows the same but only for the Tomm20 immunofluorescence to illustrate that with higher level EXD2 overexpression the mitochondrial network not only has collapsed to a condensed perinuclear mass but that this mass has become amorphous, which is more difficult to appreciate with the EXD2 fluorescent staining. For further details on these experiments see main text.

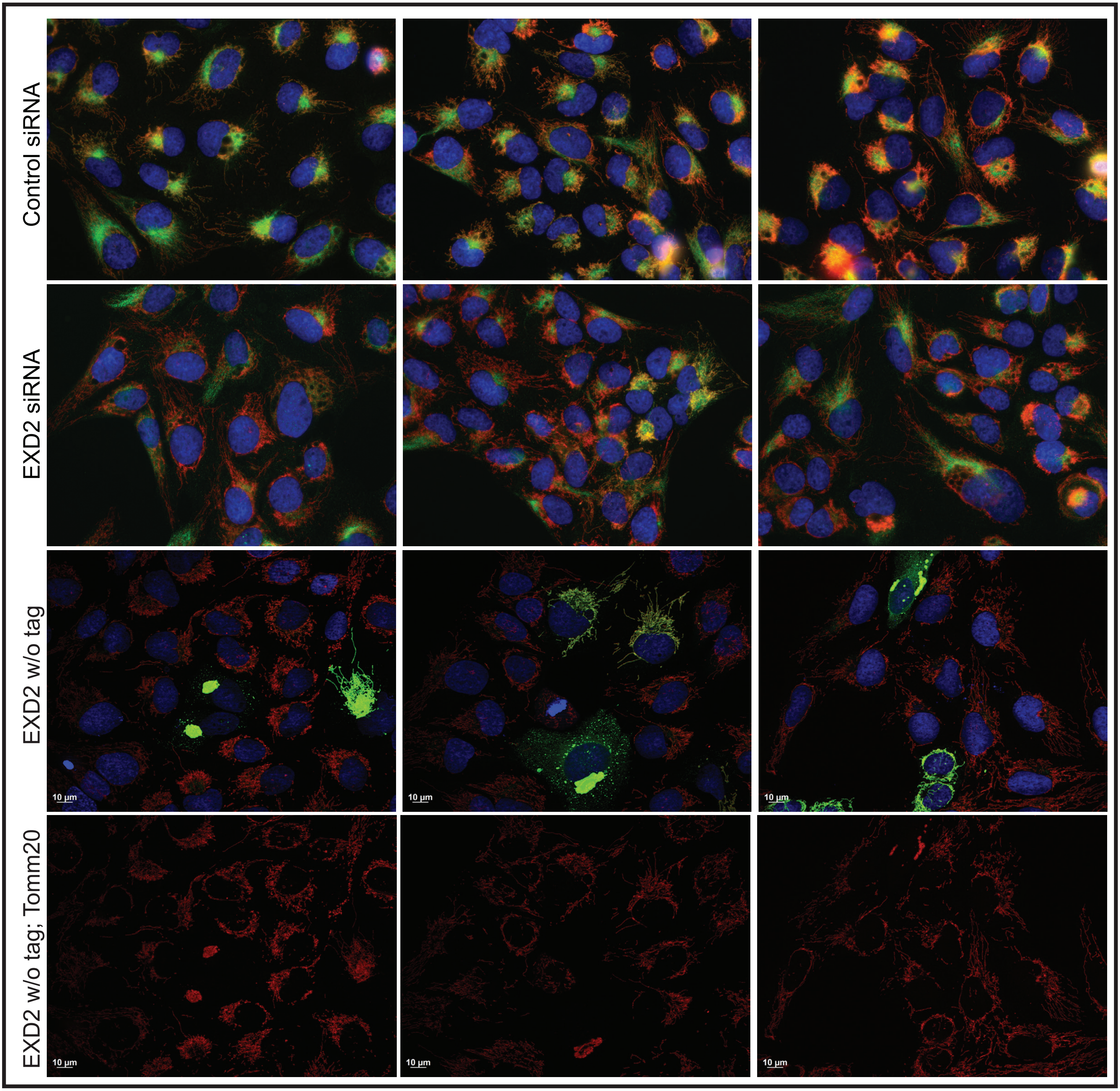

Hensen et al., Supplementary Figure 1
